# Inflammatory biomarkers as predictors of heart failure in women without obstructive coronary artery disease: A report from the NHLBI-sponsored Women’s Ischemia Syndrome Evaluation (WISE)

**DOI:** 10.1371/journal.pone.0177684

**Published:** 2017-05-19

**Authors:** Ahmed AlBadri, Kha Lai, Janet Wei, Sofy Landes, Puja K. Mehta, Quanlin Li, Delia Johnson, Steven E. Reis, Sheryl F. Kelsey, Vera Bittner, George Sopko, Leslee J. Shaw, Carl J. Pepine, C. Noel Bairey Merz

**Affiliations:** 1 Barbra Streisand Women’s Heart Center, Cedars-Sinai Heart Institute, Los Angeles, California, United States of America; 2 Biostatistics and Bioinformatics Research Center, Cedars-Sinai Medical Center, Los Angeles, California, United States of America; 3 Cardiovascular Institute, University of Pittsburgh, Pittsburgh, Pennsylvania, United States of America; 4 Division of Cardiovascular Disease, Department of Medicine, University of Alabama at Birmingham, Birmingham, Alabama, United States of America; 5 Division of Cardiovascular Disease, National Heart, Lung and Blood Institute, National Institutes of Health, Bethesda, Maryland, United States of America; 6 Division of Cardiology, Department of Medicine, Emory University School of Medicine, Atlanta, Georgia, United States of America; 7 Division of Cardiovascular Medicine, University of Florida College of Medicine, Gainesville, Florida, United States of America; University of Bologna, ITALY

## Abstract

**Background:**

Women with signs and symptoms of ischemia, no obstructive coronary artery disease (CAD) and preserved left ventricular ejection fraction (EF) often have diastolic dysfunction and experience elevated rates of major adverse cardiac events (MACE), including heart failure (HF) hospitalization with preserved ejection fraction (HFpEF). We evaluated the predictive value of inflammatory biomarkers for long-term HF hospitalization and all-cause mortality in these women.

**Methods:**

We performed a cross-sectional analysis to investigate the relationships between inflammatory biomarkers [serum interleukin-6 (IL-6), C-reactive protein (hs-CRP) and serum amyloid A (SAA)] and median of 6 years follow-up for all-cause mortality and HF hospitalization among women with signs and symptoms of ischemia, non-obstructive CAD and preserved EF. Multivariable Cox regression analysis tested associations between biomarker levels and adverse outcomes.

**Results:**

Among 390 women, mean age 56 ± 11 years, median follow up of 6 years, we observed that there is continuous association between IL-6 level and HF hospitalization (adjusted hazard ratio [AHR] 2.5 [1.2–5.0], p = 0.02). In addition, we found significant association between IL-6, SAA levels and all-cause mortality AHR (1.8 [1.1–3.0], p = 0.01) (1.5 [1.0–2.1], p = 0.04), respectively.

**Conclusion:**

In women with signs and symptoms of ischemia, non-obstructive CAD and preserved EF, elevated IL-6 predicted HF hospitalization and all-cause mortality, while SAA level was only associated with all-cause mortality. These results suggest that inflammation plays a role in the pathogenesis of development of HFpEF, as well all-cause mortality.

## Introduction

Signs and symptoms of ischemia in the absence of obstructive coronary artery disease (CAD) is highly prevalent in women and frequently associated with recurrent symptoms, repeated testing and coronary microvascular dysfunction (CMD) [1]. The pathophysiology and clinical determinants of these symptoms in such women have not been fully elucidated. However, release of vascular constricting factors and production of pro-inflammatory cytokines, cell adhesion molecules, and growth factors that, in turn, may induce inflammatory and proliferative changes in the vessel wall, has been suggested [2].

Women with signs and symptoms of ischemia and no obstructive CAD are at elevated risk for major adverse cardiac events (MACE), dominantly heart failure (HF) hospitalization [3] which we have documented to be heart failure with preserved ejection fraction (HFpEF) [4], and diastolic dysfunction [5, 6]. We have previously reported that inflammatory cytokines predict MACE but do not relate to angiographic CAD severity in women with preserved EF [7, 8]. Others have reported that elevated C-reactive protein levels correlate with symptoms and markers of myocardial ischemia in patients with chest pain and non-obstructive CAD [9]. While these observations suggest that systemic inflammation may be linked with CMD, relations to development of HFpEF have not been investigated.

Mechanistic pathways and treatment targets for HFpEF remain very poorly understood and evidence-based management strategies are lacking. Given the unexpected predominance of HFpEF hospitalization at 6-year follow-up in WISE [3, 4], we investigated the relationships between inflammatory markers [interleukin-6 (IL-6), C-reactive protein (hs-CRP), and serum amyloid A (SAA)] and longer-term adverse outcomes in this unique population of patients with signs and symptoms of ischemia with non-obstructive CAD and preserved EF as part of the Women’s Ischemia Syndrome Evaluation (WISE) study sponsored by the National Heart, Lung, and Blood Institute (NHLBI).

## Methods

### Study population and design

The WISE (ClinicalTrials.gov no. NCT00000554) design and protocol have been described in detail previously [10]. The study included 936 women referred for clinically indicated coronary angiography to further evaluate clinically stable symptoms/signs of myocardial ischemia at 4 sites (University of Alabama at Birmingham; University of Florida, Gainesville; University of Pittsburg; Allegheny General Hospital, Pittsburgh). Major exclusion criteria of the WISE study included cardiomyopathy, New York Heart Association class IV congestive heart failure, recent myocardial infarction, and significant valvular heart disease. For this analysis, participants with obstructive coronary artery disease (defined as >50% diameter stenosis in any vessel by QCA core lab) were excluded. The WISE study was approved by each institution’s review board and all participants gave written informed consent. Of the 936 women enrolled in the WISE study, a total of 456 women had non obstructive CAD and biomarker laboratory data, and 390 women had inflammatory biomarkers and follow-up information available for this analysis. Key demographic and laboratory variables were collected at baseline using standardized forms.

### Laboratory testing

Plasma sampled at baseline was frozen at -70 ^0^C and stored in a central core lab (Cedars-Sinai, LA, CA) for subsequent measurement of inflammatory markers. Levels of IL-6 was measured using a commercially available enzyme-linked immunosorbent assay kit (Quantikine hs human IL-6, R&D Systems, Minneapolis, Minn.). Levels of high-sensitivity CRP (hsCRP) and SAA were measured by a high-sensitivity method on the Hitachi 911 analyzer using reagents from Denka Seiken (Niigata, Japan). All samples were assayed within 5 years of collection at a core laboratory (Ridker P, Brigham and Women’s Hospital, Boston, Mass) using previously validated techniques [8].

### Follow-up procedures

The initial protocol-specified follow-up was conducted by experienced site nurses or physicians through direct, telephone, and/ or mail contact at 6 weeks, 1 year, and annually thereafter using a standardized scripted interview. Women were queried about symptoms, medication use, cardiovascular outcomes, hospitalizations, and diagnostic or revascularization procedures since last contact. For cases cared for at a WISE clinical center, patients’ medical records were also reviewed. The median follow-up time among surviving patients was 6 years. Subsequently, we conducted a National Death Index search for all women who were thought to be alive, and obtained additional death certificates. For this analysis, a major event (MACE) was defined as a first occurrence of all-cause mortality, nonfatal myocardial infarction (MI), nonfatal stroke, or HF-hospitalization.

### Statistical methods

Demographic and patient characteristic variables were summarized using means and standard deviations or percentages where appropriate. Distributions of inflammatory markers are known to be positively skewed, therefore, medians were used to estimate central tendencies and Spearman correlation was used to estimate correlation between markers. For time to event data, the Kaplan-Meier method and log-rank test were used to estimate the incidence rates of all-cause mortality, cardiovascular mortality and HF hospitalization. The hazard ratio for all biomarkers is reported per log change. First, we conducted univariate model to adjust for confounders including age, BMI, history of diabetes, smoking, history of malignancy, history of autoimmune disease and concomitant medications [7]. A multivariable Cox Proportional Hazards model was used after removing variables that are not significant. As a result, each outcome has different multivariable model.

## Results

### Baseline characteristics

Pertinent baseline characteristics are summarized in Table 1. The mean age was 56±11 years, most were Caucasian and postmenopausal and about half had used hormonal replacement therapy (HRT). Most had multiple CVD risk factors, including diabetes (16%), dyslipidemia (45%), family history of premature CAD (66%), smoking history (20%), obesity (mean BMI 29.9±6.8) and hypertension (mean systolic blood pressure 136±21). Overall, 55% of the women had hs-CRP values >0.3 mg/dL, and 21% had SAA >1.0 mg/dL, values that are considered abnormally high [11, 12].

**Table 1:** Baseline characteristics (n = 390).

Age (mean±SD)	56±11
CVD risk factors (%)	
• Diabetes • History of dyslipidemia • Family history of premature CAD • Current smoking • BMI (kg/m^2^) (mean±SD) • Systolic blood pressure (mmHg)(mean±SD)	• 16 • 45 • 66 • 20 • 30±7 • 136±21
Chronic kidney disease (%)	3
History of malignancy (%)	11
Depression (%)	27
History of autoimmune disease (%)	9
Medications (%)	
• ACEI • ARB • Beta-blockers • Diuretics • Vasodilators • Aspirin • Corticosteroid • HMG-Co reductase inhibitors	• 21 • 3 • 33 • 25 • 7 • 51 • 4 • 20
Inflammatory biomarkers level (median)	
• IL-6 • hs-CRP* • SAA	• 2.8 pg/mL (Range: 0.43–33.4 pg/mL) • 0.37 mg/dL (Range: 0.02–16.4 mg/dL) • 0.54 mg/dL (Range: 0.08–73 mg/dL)

*Level of <0.1 mg/dL is considered low risk for atherosclerosis.

CAD: Coronary artery disease, CVD: cardiovascular disease, BMI: body mass index, ACEI: angiotensin converting enzyme inhibitors, ARB: angiotensin receptor blockers, IL-6: interlukin-6, hs-CRP: high sensitivity C-Reactive protein, SAA: serum amyloid A.

### Adverse outcomes and inflammatory biomarkers

Among the 390 women with available follow-up data, 106 (23%) had a MACE over a median of 6 years follow up. These events included 45 deaths (11%), 28 of which (62%) were cardiovascular related causes. There was a total of 14 HF hospitalizations (3.5%), 19 nonfatal MI (4.9%), and 24 nonfatal stroke (6.2%). We observed a significant increase in all-cause mortality associated with IL-6 and SAA, while there was no significant association between hs-CRP and mortality (Table 2). There was also a significant association between IL-6 and HF hospitalization (Table 3).

**Table 2:** Adjusted relative risks (as continuous) for all-cause mortality from multivariable Cox regression analysis* (n = 390).

Biomarker	HR (95% CI)	p-value
IL-6	1.8 (1.1–3.0)	0.01
hs-CRP	1.1 (0.8–1.7)	NS
SAA	1.5 (1.0–2.1)	0.04

*adjusted for age, smoking history, diabetes, and the use of statins.

HR: hazard ratio per unit (log-scale) increase, CI: confidence interval, NS: non-significant, IL-6: interleukin-6, hs-CRP: high sensitivity C-reactive protein, SAA: Serum Amyloid A.

**Table 3:** Adjusted relative risks (as continuous) for HF hospitalization from multivariable Cox regression analysis* (n = 390).

Biomarker	HR (95% CI)	p-value
IL-6	2.5 (1.2–5.0)	0.04
hs-CRP	1.4 (0.9–2.3)	NS
SAA	1.7 (1.0–3.0)	NS

*adjusted for age, smoking history, diabetes, and the use of statins.

HR: hazard ratio per unit (log-scale) increase, CI: confidence interval, NS: non-significant, IL-6: interleukin-6, hs-CRP: high sensitivity C-reactive protein, SAA: Serum Amyloid A.

**Fig 1:**
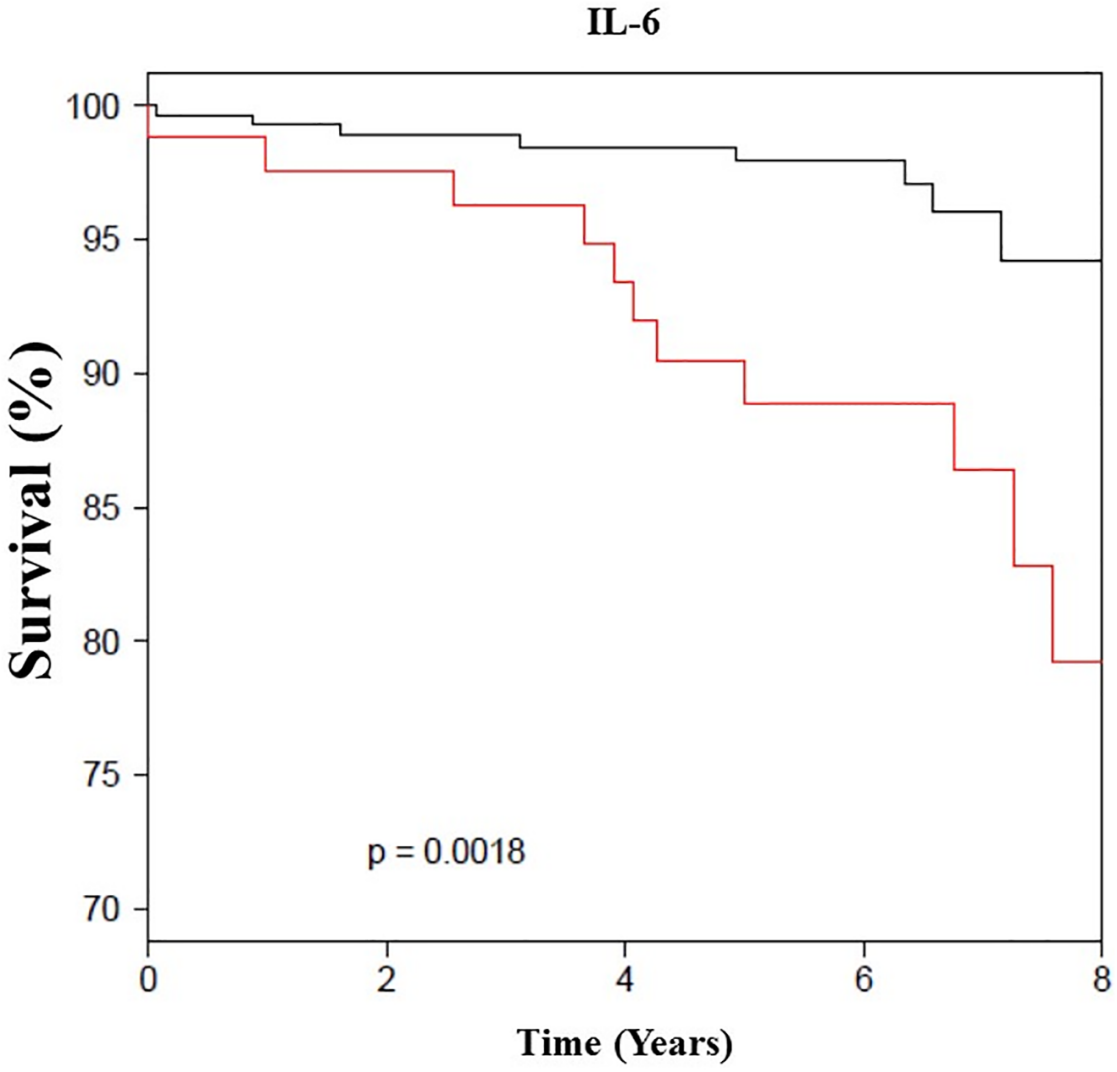
All-cause survival curves by IL-6 level (N = 390). The black line indicates IL-6 level <4.79 pg/mL and the red line is when IL-6 ≥4.79 pg/mL among women with signs and symptoms of ischemia, no obstructive CAD and preserved EF (median follow up of 6 years).

**Fig 2:**
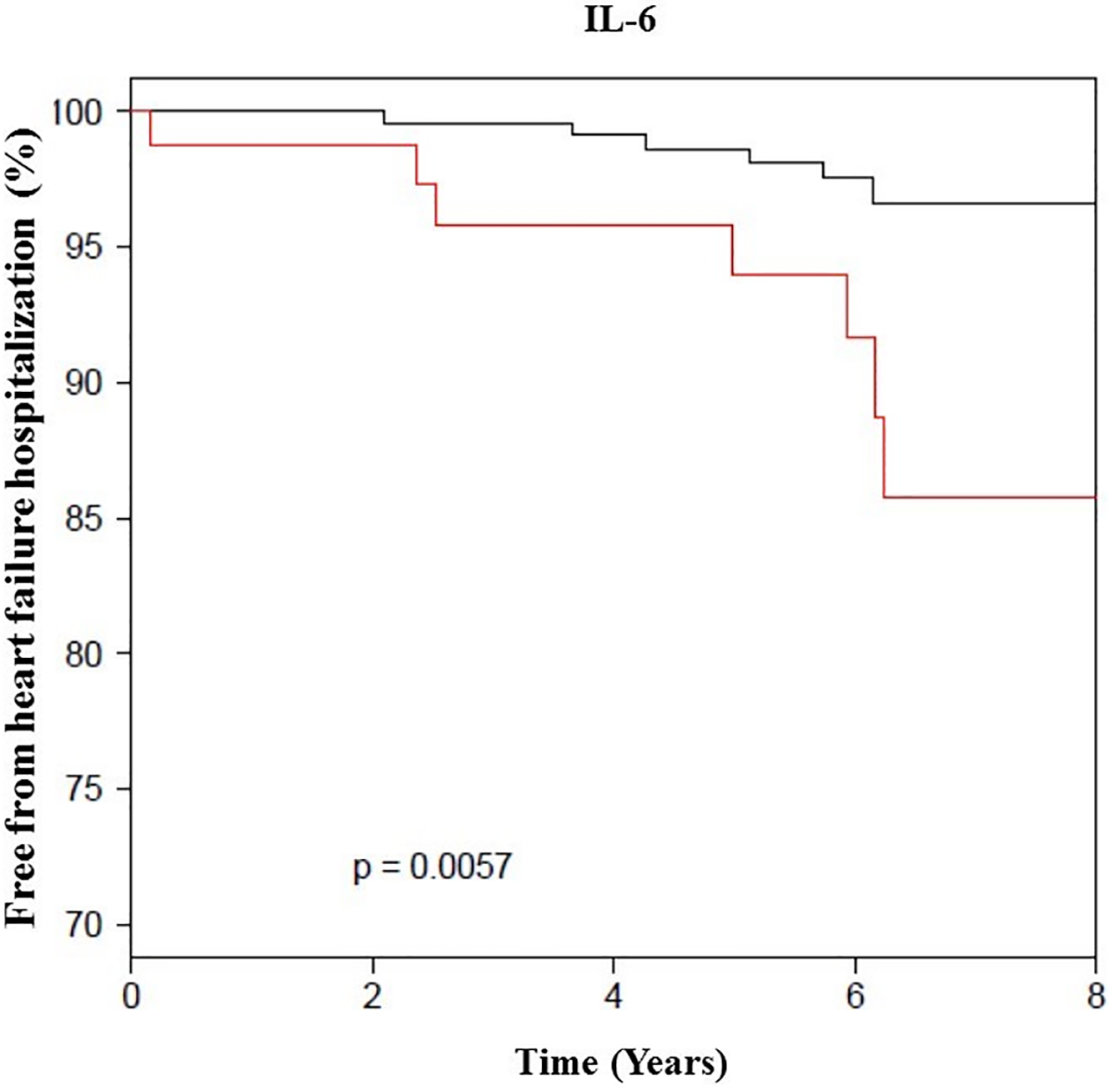
HF hospitalization by IL-6 level (N = 390). The black line indicates IL-6 level <4.79 pg/mL and the red line is when IL-6 ≥4.79 pg/mL among women with signs and symptoms of ischemia, no obstructive CAD and preserved EF (median follow up of 6 years).

Each individual inflammatory biomarker was then grouped into quartiles and in combination to assess their relative contribution to mortality. When comparing IL-6 and SAA in their highest quartile (>4.79 pg/ml, >0.9 mg/dL, respectively) to the lowest quartile, there was significant increase in all-cause mortality AHR (4.2 [1.32–13.2], p = 0.02; 3.6 [1.3–10.3], 0.02), respectively (Fig 1). Among the 42 women who had IL-6, hs-CRP and SAA levels in their upper quartiles, 8 (20%) died compared to only one (3%) of 27 women who had all the three biomarkers in their lower quartile. Additionally, a level of IL-6 greater than 4.79 mg/dL was also highly predictive of HF hospitalization during the follow up period (Fig 2).

## Discussion

We observed in this unique population of women with signs and symptoms of ischemia, non-obstructive CAD and preserved EF at enrollment, IL-6 was the strongest predictor of HF hospitalization in these women compared to hs-CRP and SAA level, especially in the highest quartile (>4.79 pg/mL). In addition to IL-6 was associated with increase mortality, SAA was linked to high mortality as well which we reported previously [7]. Notably, IL-6 predicted *mortality and HF hospitalization* during follow up, independent of cardiovascular risk factors and comorbidities, consistent with the epidemic of HFpEF which is now a major contributor to CVD mortality [3, 4]. Previously, we found IL-6, hsCRP and SAA are predictors of CVD in women, however the prior report included women with obstructive and non-obstructive CAD with shorter follow-up [7, 8]. Although plasma levels of these inflammatory markers are known to be elevated in proportion to the degree of functional disability and left ventricular dysfunction, to our knowledge, no prior study has evaluated whether inflammatory biomarkers predict the development of HF among women with normal ventricular function and non-obstructive CAD.

The measurement of inflammatory markers for risk stratification of both primary and secondary prevention of cardiovascular disease has been the focus of numerous prior investigations. These efforts have been motivated by observations that inflammation is a key process in the pathophysiology of atherosclerosis, the development of acute cardiovascular syndromes, and heart failure [7]. Not only have inflammatory markers been found to be significant predictors of cardiovascular disease in asymptomatic women [13], they also correlate with clinical activity in patients with ischemic symptoms and non-obstructive CAD [9]. In an analysis from Framingham Heart Study, the role of elevated levels of inflammatory cytokines, notably IL-6, was assessed and associated with an increased long-term risk of HF development in previously asymptomatic patients [14]. These data are congruent with our findings and our study extends this finding to symptomatic women. Furthermore, baseline LV function was not reported in the Framingham Heart Study analysis, so it is possible that the study may have identified patients with subclinical LV dysfunction. We believe this observed relationship between IL-6 and HF warrants further investigations.

A potential mechanism to understand our observed association may be association between elevated levels of inflammatory cytokines and development of HF may include atherosclerosis, endothelial and microvascular dysfunction progression [15, 16]. Inflammatory cytokines cause modulation of matrix metalloproteinases, as well as platelet adhesion and aggregation, both of which have effect on plaque formation, plaque destabilization and rupture with proximal and distal vessel occlusion and embolization [17, 18]. IL-6 is the main inducer of hepatic production of acute-phase proteins, including hs-CRP and SAA. Both play a role in plaque formation [2] and are associated with biologic and environmental risk factors for cardiovascular events, including components of the metabolic syndrome (obesity, insulin resistance, diabetes, hypertension, and low HDL cholesterol levels), and lifestyle factors, such as smoking, abstinence from alcohol, and physical inactivity [19–21]. In addition, hs-CRP has a role in atherosclerosis by down-regulating nitric oxide release from endothelial cells [22–24] and stimulating endothelin-1 and IL-6 secretion, resulting in increased expression of adhesion molecules, stimulating monocyte chemoattractant protein-1, and facilitating macrophage low-density lipoprotein uptake [23, 24]. Others have also suggested that SAA modulates HDL metabolism and may be involved in diminishing its athero-protective effect [24].

A variety of inflammatory markers have been previously identified as having a potential role in the progression of HF. We found that in this unique population of women with symptomatic ischemia, non-obstructive CAD and preserved EF at baseline, serum IL-6 was associated with an increased risk of HF hospitalization in a continuous fashion without evidence of a threshold. The mechanism is not well understood in our cohort, however elevation of such markers may be a driver or a reaction to the pathophysiological processes of progressive ventricular remodeling. Experimental data have implicated a causative role of IL-6 in cardiomyocyte hypertrophy and apoptosis [25, 26], adverse LV remodeling [27], and progression to HF [28]. IL-6 also exerts a negative inotropic effect on cardiac myocytes by augmentation of nitric oxide and peroxynitrite formation [29] and by inhibition of sarcoplasmic reticulum Ca-ATPase (SERCA2). Overall, these data suggest that future prospective evaluation of inflammatory biomarkers for clinical prognostic utility and as a mechanistic pathway for HF progression would be important.

### Strengths and limitations

Strengths of our study include a prospective, multicenter design and core laboratory masked assessments of inflammatory markers and coronary angiograms with long subject follow-up of 6 years. This allowed exclusion of obstructive CAD to investigate development of HF hospitalization in a unique population of women with signs and symptoms of ischemia, without obstructive CAD and preserved EF. A potential limitation is the highly selective study population consisting of middle age predominantly Caucasian women with suspected myocardial ischemia referred for specialized centers for clinically indicated coronary angiography. Thus our results may not be generalizable to other populations of women or to men. In addition, there were small number of events during the follow-up period. Furthermore, diagnosis of HF was defined as admission for HF, which was validated in a representative sample from one WISE site using patient hospital records [5]. Thus, data about patients with milder forms of HF, which could be managed in an outpatient basis, were not collected. Lastly, 7% of our cohort was lost to follow-up, although this is less than what had been reported to pose serious impact on the results [30]. We performed statistical comparison of baseline characteristics between patient with and without available follow-up data (lost to follow-up). There was no difference between the two groups.

## Conclusions

In women with signs and symptoms of ischemia, non-obstructive CAD and preserved EF, the inflammatory biomarker IL-6 was predictive of HF-hospitalization and all-cause mortality, while SAA predicted all-cause mortality. These results suggest that vascular inflammation may be a mechanistic pathway and treatment target for HFpEF, which is a rising epidemic contributing to CVD morbidity and mortality which predominantly impacts women.
